# The human gut phageome: composition, development, and alterations in disease

**DOI:** 10.3389/fmicb.2023.1213625

**Published:** 2023-07-05

**Authors:** Yingying Zhang, Ran Wang

**Affiliations:** Key Lab of Food Quality and Safety of Jiangsu Province-State Key Laboratory Breeding Base, Institute of Food Safety and Nutrition, Jiangsu Academy of Agricultural Sciences, Nanjing, China

**Keywords:** phage, phageome, microbiome, virome, gut

## Abstract

The human gastrointestinal tract is colonized by a large number of microorganisms, including bacteria, archaea, viruses, and eukaryotes. The bacterial community has been widely confirmed to have a significant impact on human health, while viruses, particularly phages, have received less attention. Phages are viruses that specifically infect bacteria. They are abundant in the biosphere and exist in a symbiotic relationship with their host bacteria. Although the application of high-throughput sequencing and bioinformatics technology has greatly improved our understanding of the genomic diversity, taxonomic composition, and spatio-temporal dynamics of the human gut phageome, there is still a large portion of sequencing data that is uncharacterized. Preliminary studies have predicted that the phages play a crucial role in driving microbial ecology and evolution. Prior to exploring the function of phages, it is necessary to address the obstacles that hinder establishing a comprehensive sequencing database with sufficient biological properties and understanding the impact of phage–bacteria interactions on human health. In this study, we provide an overview of the human gut phageome, including its composition, structure, and development. We also explore the various factors that may influence the phageome based on current research, including age, diet, ethnicity, and geographical location. Additionally, we summarize the relationship between the phageome and human diseases, such as IBD, IBS, obesity, diabetes, and metabolic syndrome.

## Introduction

The human gastrointestinal tract harbors a diverse population of microorganisms, known as the gut microbiome. The gut microbiome is a complex ecosystem that consists of bacteria, archaea, viruses, and eukaryotes, mainly residing in the hindgut. According to a project on Metagenomics of the Human Intestinal Tract, there are approximately 3.3 million microbial genes in the human gut, which is 150-fold more than human genes (Qin et al., 2010). Bacteria and viruses are the most abundant members of the microbiome that account for 93% and 5.8% of total DNA, respectively (Shkoporov and Hill, 2019). The number of bacteria in the gut is estimated to be of the same order of magnitude as that of human cells, reaching 3.8 × 10^13^ and 0.2 kg total mass (Sender et al., 2016). A virus is estimated to be ~10^9^ to 10^10^ virus-like particles (VLPs) per gram of feces (Mathieu et al., 2020). The presence of such a vast amount of microorganisms in the human gut has been shown to be closely linked to the development of human health and disease. Although bacteria have been the focus of most research due to their dominance, human intestinal phages have also gained widespread attention and interest.

Phages, which are viruses capable of infecting bacteria, are thought to play an important role in the structure and function of microbial communities (Shkoporov et al., 2022a), and they constitute the majority of the gut virome. Since the discovery of phage by Twort (1915), culture-based methods have been used for the screening and quantification of phages. However, these methods are limited to identifying phages that target specific bacteria and are not suitable for exploring the majority of the gut virome. Recent advances in high-throughput sequencing technologies have made it possible to investigate viromes based on metagenomics. This process involves extracting the genetic material of viruses from samples containing host cells and bacteria, sequencing the total viral DNA and RNA, and identifying and annotating the viral sequences for species and function. The phageome refers to the total community of phage populations, along with eukaryotic viruses that make up the virome. As the ways of mapping and describing intestinal microecology become increasingly sophisticated, the phageome role in health is becoming clear.

In this review, we summarize the composition, structure, and development of the human gut phageome and the factors shaping it due to our current understanding, and we also discuss the relationship between the phageome and human diseases.

## Composition and structure of the gut phageome

The human gut phage population is highly diverse and varies in its viral structure. Phageome is composed of either DNA or RNA, which can exist in either double-stranded or single-stranded form. Phages are categorized into four types based on their genome type: double-stranded (ds)DNA, single-stranded (ss)DNA, double-stranded RNA (dsRNA), or single-stranded RNA (ssRNA) (Ackermann, 2009). The dominant phages in the human gut are dsDNA and ssDNA phages. The family of dsDNA phages encompasses *Myoviridae, Podoviridae, Siphoviridae, Ackermannviridae, Corticoviridae, Tectiviridae*, and *Plasmaviridae*. The family of ssDNA phages is mainly composed of *Microviridae* and *Inoviridae* (Dion et al., 2020). The genetic material of phages is wrapped in a capsid of polyhedral, pleomorphic, or tailed shape. *Microviridae, Corticoviridae, Tectiviridae, Leviviridae*, and *Cystoviridae* are common polyhedral shape, *Plasmaviridae* is pleomorphic shape, and *Caudovirales, Siphoviridae*, and *Podoviridae* are tailed shape (Ackermann, 2009). Although sequencing technologies play a key role in delivering information unobtainable by culture-based methods, the majority of the data obtained through the macrogenome dataset cannot be aligned with the known sequences, which constitutes the viral dark matter. Evidence confirms that viral dark matter sequences can comprise 40% to 90% of the sequence, depending on the sample (Krishnamurthy and Wang, 2017). Thus, many sequence-based virome studies only analyze a small fraction of known phage sequences, which excludes viral dark matter from the analysis. This approach can lead to significant implications for the conclusions drawn from these studies, as changes in the known fraction may not accurately reflect changes in the entire virome. Traditional classification of phage by the International Committee on Taxonomy of Viruses (ICTV) was mainly based on its virion morphology, and the accuracy has been questioned. For example, phages P22 and lambda with similar genomic and functional characteristics are divided into different families based on morphology (Sutton and Hill, 2019). With the increasing number of phage genomes, genome-based taxonomy has been used to classify phages. In 2020, ICTV proposed a new 15-rank viral taxonomic classification system that classifies viral nucleic acid sequences obtained from metagenomic data to adapt to the genetic diversity of viruses (International Committee on Taxonomy of Viruses Executive Committee., 2020).

*Caudovirales* and *Microviridae* are the main members of the human gut phage community (Manrique et al., 2017). Microscopic observation revealed that *Caudovirales* was the predominant phage, and they have a dsDNA genome and are characterized by icosahedral capsids and a tail. *Caudovirales* order can be divided into three families, namely *Siphoviridae, Podoviridae*, and *Myoviridae*, based on the characteristics of the tail. The *Siphoviridae* (genome size of 39–43 kb) are characterized by a long, non-contractile, flexible tail, the *Podoviridae* (genome size of 16–18 kb) have a short tail, and the *Myoviridae* (genome size of 127–140 kb) have a long, contractile tail (Becker, 2018; Shkoporov and Hill, 2019). The tail and associated caudal fibers form a device that not only defines the targeting specificity of the viral particles but also increases the efficiency of infection. The protein capsid size of tailed phages is typically between 45 and 185 nm, which is related to their genomic size. *Microviridae* family has ssDNA (genome size of 4–7 kb) and icosahedral capsid, and most of them have always been considered exclusively lytic (Dion et al., 2020).

*CrAssphage* was discovered in 2014 by analyzing published information on viral metagenomes in human feces (Dutilh et al., 2014). The *crAssphage* is the most abundant virus in the human gut, which accounts for 90% of the sequences in the gut virome. It has a dsDNA genome and was predicted to mainly infect *Bacteroides spp*. The genome of *crAssphage* is ~100 kb, 80% of which encode predicted proteins with no significant similarity to available protein sequences (Yutin et al., 2018). Initially, genome analysis of *crAssphage* was unable to find any related phages or fully explain the function of most genes present. However, with the use of advanced methods and extensive databases, the subsequent reanalysis was successful in identifying a comprehensive collection of crAss-like phages (Koonin and Yutin, 2020). In 2018, Guerin et al. identified 249 crAss-like phage genomes from 702 human fecal phageome data, expanding the known information on crAss-like phage genomes. These crAss-like phages could be classified into four subfamilies and ten candidate genera, showing a high degree of diversity (Guerin et al., 2018). This study largely fills a gap in the paucity of genomic data and demonstrates the abundance and diversity of crAss-like phages in the human gut. CrAss-like phage has a unique genomic profile, and the genomes of one particular crAss-like phage family are nearly twice as large as the *crAssphage* genome, ranging from 145 to 192 kilobases. These phages contain a high density of self-splicing introns and inteins (Yutin et al., 2021).

Phages make up the majority of the human gut virome. The Gut Virome Database constructed from 1,986 individuals metagenomes indicated that phages account for 97.7% of the gut virome, while eukaryotic viruses and archaeal viruses account for 2.1% and 0.1%, respectively (Gregory et al., 2020). Although the structure of the phageome may vary with age, diet, ethnicity, and geography, most current studies consider order *Caudovirales* and family *Microviridae* to be the dominant populations in the phageome. An investigation of the structure of healthy adult's gut virome revealed that the virome has a dominated order *Caudovirales* (crAss-like phages, Siphoviridae, Myoviridae, and Podoviridae with relative abundance of 21.2%, 12.7%, 8.3%, and 5.0%, respectively), followed by *Microviridae* family (31.0%), *Inoviridae* (0.1%), and others (Shkoporov et al., 2019). While eukaryotic viruses, such as large nucleocytoplasmic DNA viruses (NCLDVs), as a monophyletic group of viruses infect eukaryotes, their proportions were found < 1% (Kim et al., 2011). Compared to phages, NCLDVs have larger genomes and viral sizes, and they are the largest viruses known today (Ku et al., 2020).

## The spatial distribution characteristics of phageome in gut

**Figure 1:**
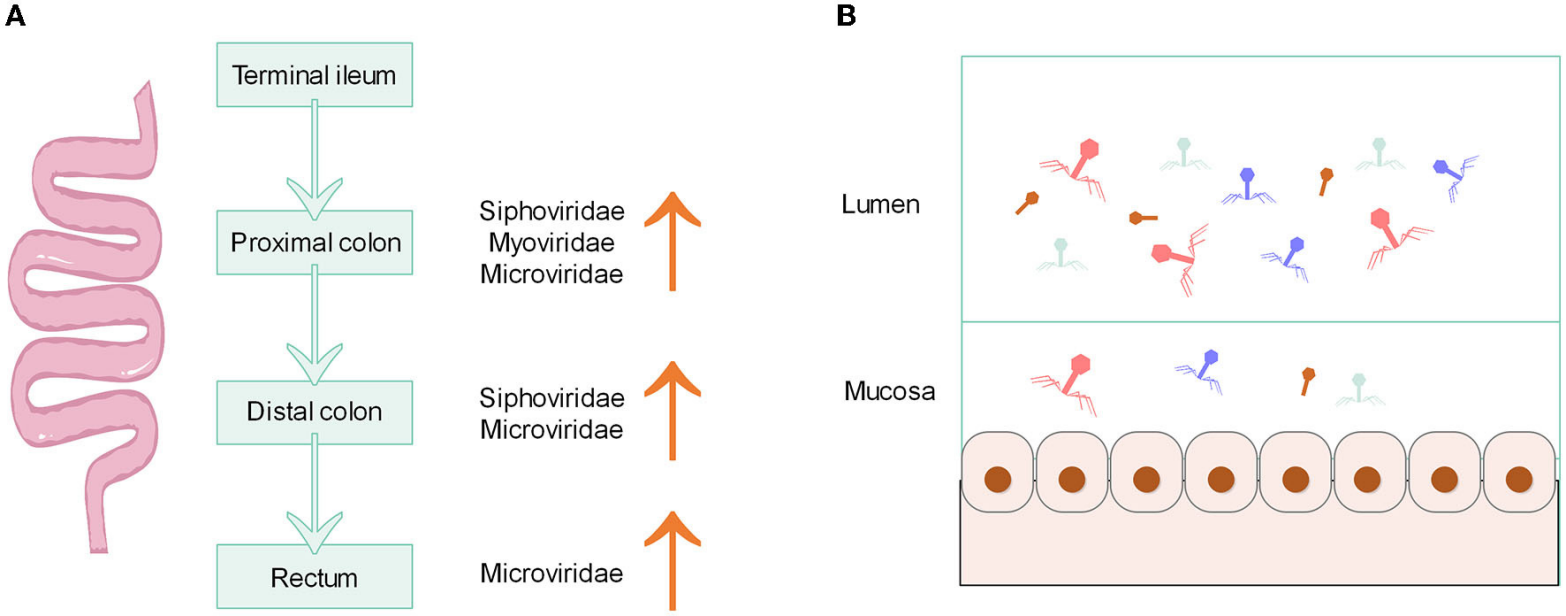
**(A)** Characteristics of phage distribution along the longitudinal axis of the primate gut. **(B)** Density of phage distribution on the horizontal axis of the gut.

The composition of microbiota in the gastrointestinal tract can potentially indicate the unique physiological properties of a specific region. The density and species of microorganisms are influenced by various factors such as chemical, nutritional, and immunological gradients (Thursby and Juge, 2017). Research conducted on both humans and animals has demonstrated that the bacterial microbiome displays distinct habitat characteristics along the vertical and horizontal axes of the gut (Tropini et al., 2017; Zhang et al., 2018; Sheth et al., 2019), while the gut biogeographic variation of phages is much less well-understood. Possibly similar to the distribution characteristics of bacteria, there exists variation in the structure and number of phages from the proximal to the distal of the gut. Using primates as animal models, samples from different parts of the intestine (terminal ileum, proximal colon, distal colon, and rectum) were taken for analysis (Figure 1A), and it was found that the abundance of phage varied between different sites. The virome of the proximal colon had higher relative abundance sequences of *Microviridae, Myoviridae*, and *Siphoviridae* compared to that of the terminal ileum. Similarly, the distal colon exhibited a higher relative abundance of *Microviridae* and *Siphoviridae*, and the rectum had a higher relative abundance of *Microviridae*, compared to the terminal ileum (Zhao et al., 2019). A comprehensive biogeographical analysis of viruses was performed in the gastrointestinal tract of two representative mammals, namely the domestic pig and the rhesus macaque by metagenomics. The large intestine of both mammals had higher levels and diversity of phages compared to the small intestine, although some viruses and phages were ubiquitous both proximal and distal. The large intestine of pig is dominated by the tailed phage *Caudoviricetes*, while rhesus macaque is dominated by *Microviridae*. The small intestine was found to be colonized by a relatively even mixture of phages and eukaryotic viruses. The majority of phage biomass and diversity was found in the colon, which can be attributed to the dense community of bacterial hosts in that particular area (Shkoporov et al., 2022a). These results suggest that the distribution of phages in the longitudinal axis of the gut is similar to that of bacteria, with higher phage density and diversity distally than proximally.

On the horizontal axis of the gut, the lumen and mucosa also possess different structures of phages. The mucus layer that covers the intestinal epithelium is one of the main ecological locations where bacteria and phages reside. The phage becomes more concentrated in mucus due to the binding interactions that occur between the mucin glycoproteins and the Ig-like protein domains on the capsid of the phage (Barr et al., 2013). There is evidence that the load and diversity of phages (class *Caudoviricetes*, family *Microviridae* and others) in the mucosa are lower than those in the lumen (Figure 1B). Total viral loads of lumen exceed 10^9^ genome copies g^−1^ contents. The viral load of the mucosa is two orders of magnitude lower than that of the lumen (Shkoporov et al., 2022a). Moreover, the mucosa has a unique virome structure, which has a higher proportion of crAss-like phages compared to stool samples (Yan et al., 2023). According to the model of “piggyback-the-winner” to explain the spatial structure and dynamics of the phage population in the lumen, the lumen may have a higher density of lysogenic rather than lytic phages, along with a lower virus-to-microbe ratio (Shkoporov and Hill, 2019), because the “piggyback-the-winner” model assumes that high microbial densities favor lysogenic rather than lytic phage replication (Gregory et al., 2020). In the inner mucus layer with low microbial density, the interaction between phage and bacteria is more suitable to be explained by the “kill-the-winner” model, which makes a density-dependent switch to a lytic cycle in temperate phages (Shkoporov and Hill, 2019). However, it remains unclear whether the distribution of phages has the same pattern of distribution across the horizontal axis as that of bacteria because virome structure and diversity are not correlated with bacterial communities, and viruses can also shape bacterial communities (Bonilla-Rosso et al., 2020).

## Age, diet, ethnicity, and geographical location are the main factors influencing the gut phageome

The establishment of normal gut microbiota is important for healthy development, especially the development of gut phageome in infants. The viral particles in infants' feces are typically rare or completely absent at birth. By 1 week of life, there is a significant surge in the number of phages found in their fecal samples (Beller et al., 2022). The number of VLP per gram of feces grows to 1 billion at 1 month of age, close to that of adults, mainly due to prophages induced by early colonizing pioneer bacteria. By 4 months of age, the number of infectious viruses in infants begins to increase. The composition of phageome during this stage is largely influenced by feeding practices. Breastfeeding has been shown to have a significant inhibitory effect on many viruses and can increase the number of temperate phages from the *Bifidobacterium* and *Lactobacillus* genera (Liang et al., 2020). Therefore, the source of neonatal intestinal phage inoculation may be through pioneer bacteria induction or breastfeeding. For example, crAss-like phages are rarely detected in newborn infants and become increasingly common after the first month of life. The genome sequences of crAss phages in mothers and infants are almost identical, indicating that the transmission of crAss-like phages from mother to infant is vertical. Although the diversity of typical crAss phage acquired by infants is less compared to that of mothers, its diversity gradually increases after colonization (Siranosian et al., 2020). The delivery mode may also contribute to differences in the structure of phageome in newborns. Newborns delivered by cesarean section reportedly lack *Bacteroides* in their gut. Meanwhile, infants by cesarean section also tested negative for prototypical *crAssphage*, whereas infants born vaginally tested positive for prototypical *crAssphage* (Siranosian et al., 2020). This is in line with the description that *crAssphage* mainly hosts *Bacteroides* species.

**Figure 2:**
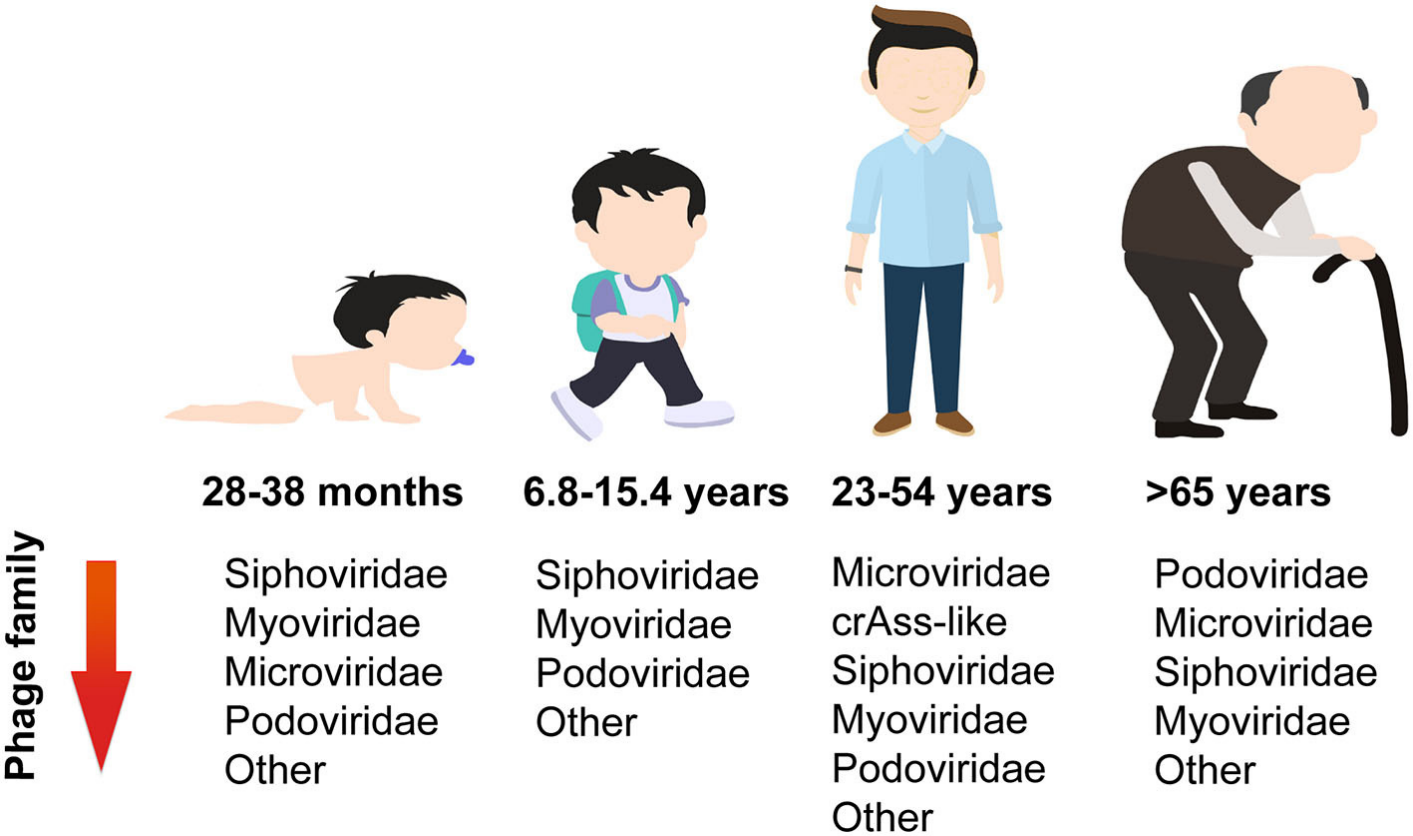
Changes in abundance of phage families with age.

The structure of the gut phageome varies with age (Figure 2). The dominant phage families during childhood are *Siphoviridae, Myoviridae*, and *Microviridae*. Infants aged 14–23 months have a higher proportion of *Podoviridae* (28%), *Myoviridae* (14%), and *Microviridae* (6%), whereas older infants aged 28–38 months had greater proportions of *Myoviridae* (21%) and *Microviridae* (18%). Meanwhile, both younger and older infants have almost 50% abundance of *Siphoviridae* (Khan et al., 2020). In the case of children (6.8–15.4 years), their dominant phage families were *Siphoviridae* (41%), *Myoviridae* (25%), and *Podoviridae* (11%) (El Mouzan et al., 2023). The adult phage community is dominated by order *Caudovirales*, which includes crAss-like phages, and families *Siphoviridae, Myoviridae*, and *Podoviridae*. Their cumulative relative abundances of reads were reported as 21.2%, 12.7%, 8.3%, and 5.0%, respectively. *Microviridae* is another dominant family with 31% relative abundance, while other families, such as *Inoviridae, Circoviridae*, and *Genomoviridae*, have a lower percentage (Shkoporov et al., 2019). Although both the phageome of children and adults are primarily composed of members from the order *Caudovirales* and family *Microviridae*, there is a shift in abundance as individuals age. Specifically, the abundance of *Caudovirales* members decreases, while the abundance of the *Microviridae* family increases. For those over 65 years of age, *Podoviridae* is the dominant family, followed by *Microviridae, Siphoviridae*, and *Myoviridae* (Gregory et al., 2020).

It has been proposed that the gut phageome of all healthy children is thought to be dominated by temperate phages. The proportion of temperate phages in the gut of 1-year-old children was found to be as high as 73% (Mathieu et al., 2020), and compared to older children, young children had more temperate phages (Khan et al., 2020). More than 60% of the *Escherichia coli* isolated from fecal samples of 1-year-old infants will spontaneously release temperate phages, and the host range of these temperate phages on *Escherichia coli* is very narrow. Conversely, the virulent phage host range is much larger, which may help them survive in the ecosystem and play a role in the dynamics of gut microbiota (Mathieu et al., 2020). A previous study conducted on healthy adult feces samples revealed that 90% of isolated coliphages were temperate (Furuse et al., 1983). However, a more recent study used metagenomics to analyze the fecal virome composition in healthy adults and found that the gut virome is highly individual and stable. The gut is predominantly inhabited and persistently affected by virulent crAss-like and *Microviridae* phages (Shkoporov et al., 2019).

Gut virome diversity is age-dependent in healthy individuals, as reported recently. In total, 27 infants (0–3 years old), 11 children (3–18 years old), 93 adults (18–65 years old), and 20 older adults (>65 years old) were included in the gut virome analysis. Throughout the human lifespan, both infants and adults exhibit the highest levels of viral richness. In fact, adults have significantly higher levels than children, while the elderly have significantly lower levels than adults. The phage family *Siphoviridae* shares the same trends with overall viral richness, and this may be because most viruses are temperate phages, belonging to the *Siphoviridae*. Interestingly, the richness of *Microviridae* sequenced showed a modest peak during infancy, followed by a drop during childhood, and then a gradual increase throughout the remainder of the lifespan (Gregory et al., 2020).

Diet is one of the important factors affecting the structure of the gut microbiome. Until now, little attention has been paid to the effect of diet on phagome. Nevertheless, several reports have found that diet can alter the phageome. Preliminary data have shown the contribution of diet in inducing bacteriophage production. For example, the consumption of a diet high in fructose has been shown to increase the production of phage in the intestinal symbiotic bacteria known as *Lactobacillus reuteri*. The main mechanism is that fructose and short-chain fatty acids can activate the ACK signaling pathway, which is related to the production of acetic acid, which can trigger a bacterial stress response that promotes phage production (Oh et al., 2019). Dietary fat also induces changes in the gut phageome. The study conducted on mice as an animal model revealed that a shift from a normal diet to a high-fat diet can alter the diversity and abundance of gut phageome. This shift was primarily observed as an increase in the abundance of *Microviridae* family and a decrease in the abundance of *Siphoviridae* (Schulfer et al., 2020). Owing to a longitudinal analysis of the gut virome in healthy adults by a gluten-free diet intervention, it was discovered that the individual virome remained stable at the family level but showed greater variability at the genus and species levels. The gluten-free diet was found to increase the abundance of *crAssphage* while decreasing the abundance of *Virgaviridae*. Moreover, the impact of the dietary intervention on the gut virome was more noticeable in populations with lower initial diversity (Garmaeva et al., 2021).

The gut virome varies considerably between individuals, and this view is now confirmed by Li et al. In their study, the researchers implemented rigorous dietary control measures on various ethnic groups and discovered that ethnicity has a greater impact on their microbiome and virome than short-term dietary interventions. Specifically, they found that individual and ethnicity factors accounted for 70–88% and 2–10% of taxonomic variation, respectively, which greatly surpasses the effects of short-term dietary interventions (Li et al., 2022). In addition to inter-individual variation, the gut virome can also be influenced by factors such as geographical locations and cultural practices. However, it can be challenging to distinguish the exact impact of each of these factors (Holtz, 2020; Nishijima et al., 2022). Participants from different regions, ethnic groups, and cities or villages in China were drawn to map the virome. Among the 20 factors that affect virome variation, geographical location is the most influential. Additionally, the dietary habits of various ethnic groups are linked to specific virus types. Urbanization also plays a role in increasing inter-individual variability in the gut virome, and the duration of urban residence is connected to a range of phageome, including Lactobacillus phage and Lactococcus phage (Zuo et al., 2020). Although several studies have initially revealed the relationship between host factors and the gut virome, there are still some that remain unnoticed, such as drugs, water sources, migration, age, diet, ethnicity, and geographical location.

## Gut phageome in disease

IBD is a group of chronic inflammatory diseases that include Crohn's disease (CD) and ulcerative colitis (UC) (Strober et al., 2007). Diversity of the gut virome in IBD patients differs from that of healthy individuals. In patients with CD, there is a lower diversity in the DNA and RNA virome. Dysbiosis is also present in the virome, which includes the DNA eukaryotic Torque teno virus (TTV), disease-associated *Faecalibacterium* phage and *Escherichia phage*, and RNA tomato diet-related virus (Kong et al., 2023). Patients with UC exhibit a greater prevalence of DNA viruses, specifically *Caudovirales* phages. The diversity, richness, and evenness of *Caudovirales* are lower in UC patients compared to healthy individuals (Zuo et al., 2019). Although efforts have been made to uncover the virome characteristics of patients with IBD, most virome analyses are performed on a limited subset of known viruses. Clooney et al. grouped viral sequences into putative higher taxonomic ranks to describe the IBD entire virome variations beyond the known minority, the virulent phage core found in healthy individuals is substituted with temperate phage in IBD patients, and the increased temperate phage in IBD patients was classified as *Siphoviridae* and *Myoviridae* (Clooney et al., 2019). Most of the increased *Siphoviridae* had CRISPR hits to Firmicutes, which is consistent with the fact that IBD patients have a lower abundance of Firmicutes (Clooney et al., 2019; Chamorro et al., 2021; Yang M. et al., 2021).

The virome may be an important contributor, in cases where the gut microbiome is thought to be an important factor in causing IBS. Previous studies have established that the families *Myoviridae, Podoviridae*, and *Siphoviridae* were the most common phage community in both IBS and healthy individuals (Ansari et al., 2020; Coughlan et al., 2021). Similar to individuals with IBD, IBS patients also exhibit a lower alpha diversity of viral clusters both in the known and unknown viruses, and their beta diversity was also different from that of healthy individuals (Coughlan et al., 2021). The whole viral diversity and abundance were reduced in IBS patients (Ansari et al., 2020). In contrast to the IBD patients, the phageome composition of IBS patients was not observed to replace dominant lytic phage with temperate phage (Coughlan et al., 2021). According to research, the gut virome differs among individuals with different subtypes of IBS. Those with IBS-D have a higher proportion of *Microviridae, Myoviridae*, and *Podoviridae* species, while those with IBS-C have a higher proportion of other *Microviridae* and *Myoviridae* species (Mihindukulasuriya et al., 2021). Consistent with this finding, Coughlan et al. also found that IBS can increase some *Mimiviridae* and *Podoviridae* species, but some decrease in *Mimiviridae, Siphoviridae*, and *Podoviridae* species was also detected (Coughlan et al., 2021). Other significantly different phage families, such as *Poxviridae, Phycodnaviridae, Pandoraviridae, Adenoviridae*, and *Rudiviridae*, were less abundant in IBS (Ansari et al., 2020).

Gut virome has been reported to differ in obesity and lean subjects. A pioneering study established the relationship between viral content and obesity metabolic measures in obese and normal mice, and obese mice were found to have higher levels of viral content than normal mice (Yadav et al., 2016). There is still debate about the diversity of the virome in obese. In a recent study, the gut virome of individuals with a BMI ≥28 (classified as obese) and those with a BMI between 18.5 and 23 (classified as non-obese) were analyzed. The study found that the richness and diversity of the virome in obese individuals were significantly lower than those in non-obese individuals. This was particularly evident in obese individuals with diabetes, where the virome diversity was especially low (Yang K. et al., 2021). Meanwhile, another study focused on the gut dsDNA virome of childhood obesity and metabolic syndrome yielded different conclusions. They found that children with obesity and metabolic syndrome had higher diversity and richness of dsDNA virome (Bikel et al., 2021). In terms of the composition of the virome in obese individuals, all animal and human studies agree that *Caudovirales* order is the dominant phage in both obese and normal individuals (Kim and Bae, 2016; Bikel et al., 2021; Yang K. et al., 2021), and the high-fat, high-sucrose Western diet-fed obese mice are more likely to enrich temperate phages of the *Caudovirales* order (Kim and Bae, 2016). Beyond this, IBD also seems to be more enriched in temperate phages, so the proportion of temperate phages seems to be closely related to human health and may be biomarkers for some diseases. Moreover, phage species enriched in obese individuals include *Escherichia* phage, *Geobacillus* phage, and *Lactobacillus* phage (Yang K. et al., 2021).

There are several lines of evidence to support the views that people with diabetes have a different gut virome. Patients with obesity and type 2 diabetes have lower viral diversity than those with obesity alone, suggesting that individuals with type 2 diabetes have a unique gut virome structure (Yang K. et al., 2021). Ma et al. demonstrated for the first time an association between the gut phageome and type 2 diabetes, by comparing the gut phageome of type 2 diabetic and healthy individuals. Type 2 diabetics have a higher number of phages in the gut and an increased abundance of *Myoviridae, Podoviridae, Siphoviridae*, and unclassified *Caudovirales* families (Ma et al., 2018). This is consistent with findings on the gut viral load of obese patients, suggesting that the expansion of phage numbers may influence the development of obesity and type 2 diabetes. Few clinical studies have investigated the role of phages in the development of human type 2 diabetes. Researchers at the University of Copenhagen discovered that mice that were fed a high-fat diet gained weight at a slower rate and maintained normal blood glucose levels after receiving a fecal virome transplant (FVT) from lean mice. They also confirmed that changes in gut microbiota, induced by the FVT, may have played a role in mediating these protective effects on metabolism (Rasmussen et al., 2020). Therefore, phages may be used to treat gut microbiota-related diseases such as obesity and diabetes. The onset of type 1 diabetes is preceded by a preclinical period of islet autoimmunity. The way in which the virome may affect type 1 diabetes is that the virus causes islet autoimmunity by shaping the bacteriome. The imbalance of *Bacteroides* genus is reported in the feces of infants with early-onset islet autoimmunity, especially the low abundance of *Bacteroides vulgatus* and *Bifidobacterium bifidum*. The quantitative relationship between bacteriophage *crAssphage* and *Bacteroides* genus implies that *crAssphage* could potentially alter the gut bacteriome in a way that contributes to the islet autoimmunity (Cinek et al., 2017).

Metabolic syndrome also has been found to be linked to gut virome alterations. Similar to diseases such as IBS, obesity, and diabetes, patients with metabolic syndrome also exhibit a reduced richness and diversity in their gut virome. Meanwhile, the phages infecting *Streptococcaceae* and *Bacteroidaceae* were enriched, and the phages infecting *Bifidobacteriaceae* were depleted in metabolic syndrome patients (De Jonge et al., 2022). Previous studies of virome changes in other diseases have failed to obtain relevant marker virus clusters, while the *Roseburia* virus cluster was shown to be associated with metabolic syndrome, and *Faecalibacterium* and *Oscillibacter* virus cluster was shown to be associated with healthy individuals in this study (De Jonge et al., 2022). Recently, the role of *crAssphage* subfamilies in metabolic syndrome and obesity is presented. Metabolic syndrome patients showed a significant decrease in the diversity and richness of the crAssphage Alpha subfamily, while there was a notable increase in the Beta subfamily. A significant correlation exhibited between the decreased crAssphage abundance and the overabundance of *Bacilli*. This suggests that *Bacilli* could potentially serve as a marker in metabolic syndrome cohorts. Therefore, the loss of stability in the alpha subfamily of *crAssphages* may contribute to the metabolic syndrome (Cervantes-Echeverría et al., 2023).

## Conclusion and future perspectives

**Figure 3:**
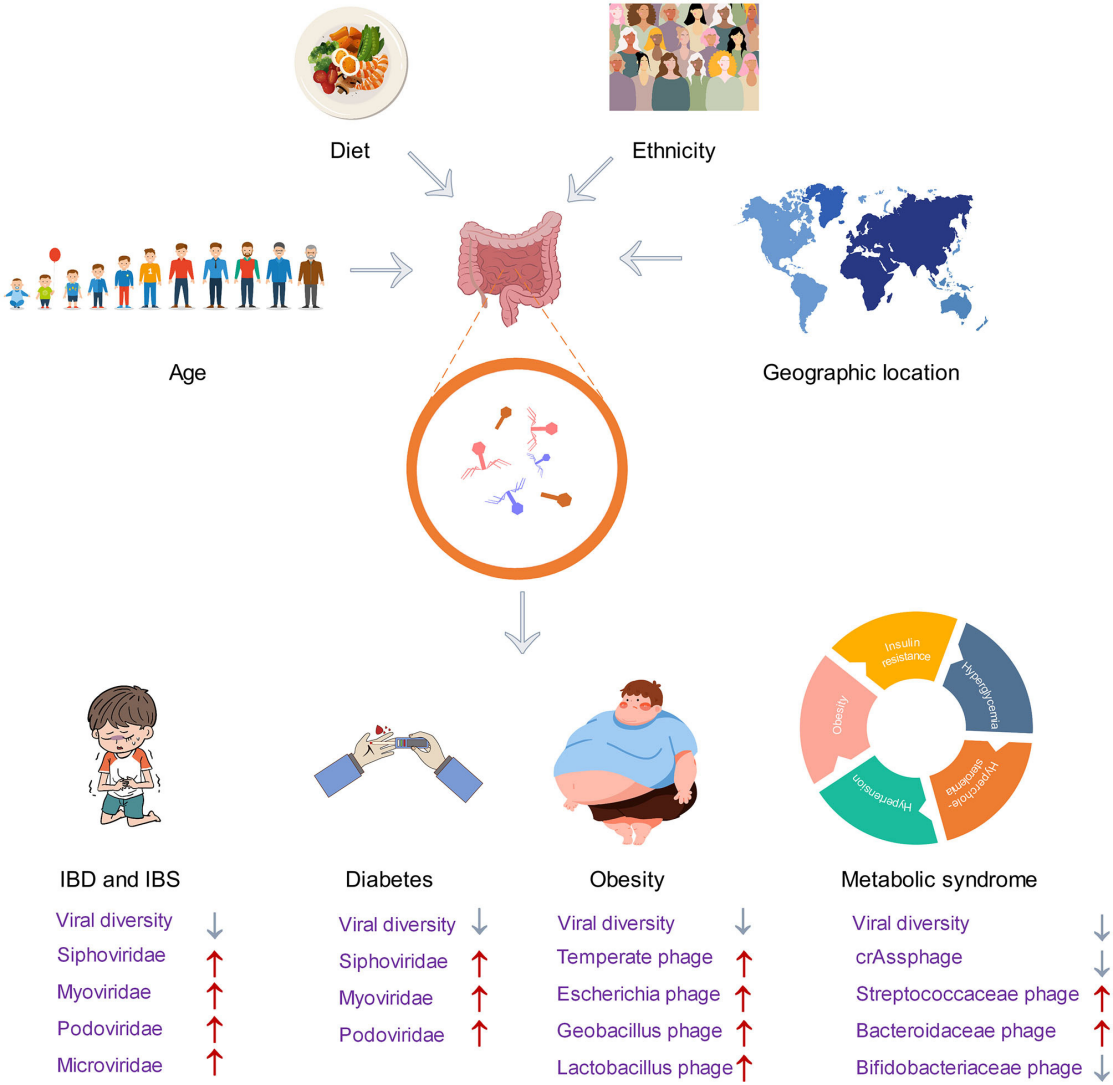
Factors affecting the gut phageome and changes in the structure of gut phageome in disease.

In the past decade, the use of viral metagenomics has significantly improved our comprehension of the unknown aspect of the gut phageome, and this has enabled researchers to better understand its taxonomic composition, development, and its role in health and disease. Sequencing analysis focused on phageome has revealed viral populations that were unnoticed before. These populations may have potential roles in interacting with the bacteria and maintaining health. Indeed, phages and bacteria are shown to be no longer simple predator–prey relationships but mutually beneficial co-existing partners (Shkoporov et al., 2022b). Growing evidence links IBD, diabetes, obesity, and other diseases to phageome changes. Viral markers for a number of diseases are gradually being identified (Cervantes-Echeverría et al., 2023; Kong et al., 2023). Figure 3 provides an overview of the main factors affecting the virome and the structural changes of the phageome in disease. With the growing problem of antibiotic resistance, phage therapy has received renewed attention (Cahan, 2023). However, phages are a class of viruses that are strictly host-dependent (Dowah and Clokie, 2018), meaning they can only be cultured and obtained after obtaining their host bacteria. It is important to note that most gut bacteria cannot be isolated and cultured *in vitro* (Lin et al., 2021). Despite having access to the genome sequence of phageome, identifying them from complex genomic data remains a challenging task.

Recent developments in the field of bacteriophage identification involve the use of new MS techniques, specifically the detection and identification of phage proteins *via* LC-MS/MS-based methods. These methods offer several advantages over other approaches as they allow for direct phage identification without the need for genetic tools (Abril et al., 2022). Therefore, the development of sequencing technologies and phage proteomics may have great potential in illuminating viral dark matter. These approaches may enhance the understanding of the taxonomic information on phageome and also facilitate the exploration of the functional potential of phage genomes.

## Author contributions

All authors listed have made a substantial, direct, and intellectual contribution to the work and approved it for publication.
